# A Preliminary Experimental Analysis of In-Pipe Image Transmission Based on Visible Light Relay Communication

**DOI:** 10.3390/s19214760

**Published:** 2019-11-01

**Authors:** Wen Zhao, Mitsuhiro Kamezaki, Kaoru Yamaguchi, Minoru Konno, Akihiko Onuki, Shigeki Sugano

**Affiliations:** 1 Graduate School of Creative Science and Engineering, Waseda University, Tokyo 169-8050, Japan; kame-mitsu@aoni.waseda.jp (M.K.); k_yamaguchi@suganos.mech.waseda.ac.jp (K.Y.); sugano@waseda.jp (S.S.); 2 Tokyo Gas Co. Ltd., Tokyo 105-8527, Japan; m-konno@tokyo-gas.co.jp (M.K.); ak-onuki@tokyo-gas.co.jp (A.O.)

**Keywords:** visible light relay communication (VLRC), image quality, pipe inspection

## Abstract

The regular inspection of underground facilities such as pipelines is absolutely essential. Pipeline leakage caused by corrosion and deformation must be detected in time, otherwise, it may cause fatal disasters for human beings. In our previous research, a robot chain system (RCS) based on visible light relay communication (VLRC) for pipe inspection has been developed. This system can basically realize the light-based transmission of control command signals and illuminance-based coordinated movement, whereas the collection and transmission approach of the pipe leakage image have not been studied. Compared with former in-pipe wireless communication techniques, VLRC can not only overcome the instability and inefficiency of in-pipe data transmission but also extend the communication range with high transmission rates. The most important feature is that it can provide a stable illumination and high-quality communication for pipe inspection robot and finally improve the energy efficiency. Hence, the aim of this article is to analyze the performance of VLRC-based image transmission in the pipe and in the future provide a high-quality, long-range, and high-efficiency image transmission for complex infrastructure inspection with RCS. The transmission systems based on two signal transmission modes analog image signal relay transmission (AISRT) and digital image frame relay transmission (DIFRT) have been proposed. Multiple experiments including the waveform test, the test of transmission features with different bit error rate (BER), and in the different mediums were conducted between these two systems. The experiment revealed that DIFRT was superior to the AISRT in terms of the relatively high-quality image transmission and reconstruction quality. It could better overcome the attenuation brought by the absorption and scattering effects and finally increased the transmission range than former communication methods. The DIFRT system could also operate at 50 kbps with relatively low BER whether in the air or water. The technique in this research could potentially provide a new strategy for implementations in the stable, effective, high-speed, and long-range image transmission of the robots in some other special environments such as tunnel, mine, and underwater, etc.

## 1. Introduction 

As important components of underground infrastructure, pipelines are designed for collecting and transporting resources such as oil, gas, water, etc. However, the pipeline network is vulnerable to losing its functionality due to internal corrosion, cracking, deformation, and manufacturing flaws. Around the world, accidents and damage resulting from pipeline leakage are often reported by the media. If the accident happens with a water pipeline, it is a waste of resources and may cause a slight environmental pollution [1]. However, if a pipeline which transports oil, gas or chemical liquids leaks it not only wastes resources, but also leads to more severe environmental pollution and possible human injury. Hence, for the safe operation and stable supply of the resource, periodic pipeline inspection and maintenance are urgently required.

Pipeline inspection tasks mostly rely on two important techniques: leakage pre-localization and localization methods. The pre-localization method refers to a preliminary external detection system based on pressure or acoustic sensors, fiber optic networks, ground-penetrating radar, etc. This method can quickly estimate the approximate location of a leakage, however, the detection accuracy is limited. In order to obtain precise leakage information, localization methods based on mobile in-pipe inspection systems are required. These systems can further detect, classify, and localize the leakages in pipelines [2,3,4,5].

Recently, several in-pipe visual inspection approaches have been widely utilized for underground pipeline network inspection and maintenance. The entire visual inspection system is usually composed of a mobile device (e.g., robot), a camera, and a lighting device mounted around the camera. When the system moves along the pipe, it can transmit the detection image to the external monitor outside the pipe. During the inspection, the captured images can be identified, classified, and located by the operator. Although this method is time-consuming and labor-intensive, most of the internal corrosion, cracking, and deformation inside the pipe can be accurately diagnosed [6,7,8].

The accurate acquisition and efficient transmission of pipeline internal image information with wireless approaches are extremely crucial for pipeline detection. However, there are still difficulties in realizing the above requirements due to the complexity of the underground pipeline environment and the attenuation effect of the transmitted signal. As the analysis in our former research, the visible light communication technique has exhibited some advantages in pipeline inspection. This article concentrates on the analysis of the in-pipe image transmission system based on visible light relay communication (VLRC) technology in order to realize reliable image transmission [9]. The transmission is carried out with two methods: analog image signal relay transmission (AISRT) and digital image frame relay transmission (DIFRT). Among them, discrete cosine transform (DCT) and inverse discrete cosine transform (IDCT) method are adopted for DIFRT. Moreover, the data frame and modulation approaches are specifically designed and implemented to increase the precision and efficiency of image transmission. The DIFRT can assist the system to reconstruct the in-pipe image information with smaller image information distortion and higher image transmission quality. It can basically satisfy VLRC-based image transmission for the pipe inspection [10].

The rest of this article is organized as follows: Section 2 introduces the related works and Section 3 analyzes the image transmission channel and quality evaluation. Section 4 and Section 5 explain the analog image signal and digital image frame relay transmission, respectively. Section 6 describes the experimental results and discussion, including the waveform test on analog/digital image signal, test of image transmission with different bit error rate (BER) or in the different mediums. In Section 7, we summarize the results and propose future works.

## 2. Related Works

Currently, signal transmission technologies based on radio frequency (RF) from the 900 MHz to 2.4 GHz and are widely used in pipe inspection. However, the signal is not stable and the most important feature is the limited signal transmission distance due to the huge attenuation in the small diameter pipe. Apart from this, some other wireless solutions based on laser techniques have been investigated, as the laser beam possesses a special feature which can be described as point-to-point transmission. For stable communication in a pipe, the high directivity of the laser transmitter and receiver is important, however, it is difficult to realize with a mobile robot system. Moreover, the size of the laser generator and the heat sink is another critical factor which limits application in small diameter pipes. Iinfrared radiation (IR) is also commonly used as a communication approach for pipe inspection robots, but it cannot avoid drawbacks such as a low transmission rate, low anti-interference capability, etc. [11].

As one type of 5G communication technique, visible light communication (VLC), has been regarded as an alternative communication method to challenge existing wireless communication, especially in complex environments such as underground, underwater, and air. The utilization of the visible light band can drastically increase the available bandwidth for wireless communication. The other advantages such as high transmission rate, high energy efficiency, and strong anti-inference capability will also make this technology more competitive than the common wireless transmission strategies.

In previous research, a robot chain system (RCS) based on VLRC and illuminated assessment for pipe inspection has been proposed. The robot system can not only achieve high-quality and low-loss communication with the external controller through the “light signal relay”, but also can detect the adjacent distance by the illuminance-based evaluation approach [12]. However, the transmission of essential information such as image through the visible light has not been realized.

There are many related studies on image transmission based on visible light. In 2013, Png et al. developed an integrated audio, video, and data transceiver system based on VLC. The real-time analog closed circuit television (CCTV) video signals and pulse-position modulated stereo audio signals could be transmitted by a white light emitting diode (LED) between a tablet and an android smartphone. Through VLC, the mobile equipment which installed visible light transceiver could access to the internet very easily [13]. In 2013, Doniec et al. developed a high-bandwidth wireless optical communication device called AquaOptical II. This system could transmit digital 1288×964 pixels images reliably through a unidirectional underwater channel of 50 m length. Besides, the excellent performance of real-time image acquisition and high-quality displaying under 200 m depth of water made it possible to have a wide range of applications such as underwater robot tele-operation and interactive remote seabed monitoring [14]. In 2016, Narmanlioglu et al. demonstrated their research results using on-board camera video transmission through a vehicle lamp. With the support of direct current biased optical orthogonal frequency division multiplexing (DCO-OFDM) technique, Narmanlioglu et al. finally realized the line of sight (LOS) inter-vehicular video transmission based on VLC with the data rate of 6.42 Mbps and lower latency than 32 ms in the experiments [15]. In 2018, Han et al. proposed and implemented an optical-acoustic hybrid network. In this network, optics provided good quality real-time video streaming while acoustic maintains a channel for the network topology and transmission control. The acoustic channel was also used for frame video delivery when the optical channel fails. Han et al. had also demonstrated robust multi-point, low power omnidirectional optical 128×96 pixels image transmission over range of 25 m at data rates up to 9600 bps between the underwater transmitter and receiver [16].

## 3. Analysis of Image Transmission Channel and Quality Evaluation

**Figure 1:**
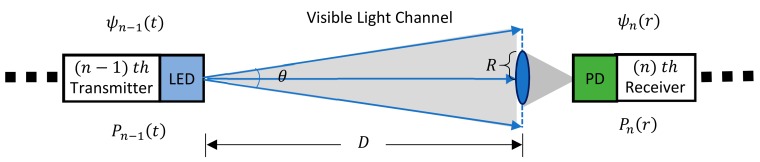
Structure of image transmission channel based on VLRC.

Like wireless signals, as illustrated in Figure 1, attenuation of optical signals also occurs in other transmission channels such as water, air, etc. The attenuation effects of the optical beam in the medium involves two completely different processes, i.e., absorption and scattering effects [17,18,19]. The absorption is an irreversible process in which the molecules in the medium absorb photons and convert them into thermal or chemical energy before they are radiated again. The scattering effect refers to the process in which photons are incident into the medium without energy conversion, and then are emitted again in a random direction. Two important parameters can reflect the absorption and scattering effects. The absorption coefficient $a\left(\lambda \right)$ and scattering coefficient $b\left(\lambda \right)$ indicate the absorptivity and scattering rate per unit length in the medium, respectively. The channel attenuation coefficient $c\left(\lambda \right)$ of light can be expressed by the following formula:

(1) $$c\left(\lambda \right)=a_{\omega }\left(\lambda \right)+a_{\gamma }\left(\lambda \right)+a_{\varphi }\left(\lambda \right)+b_{\omega }\left(\lambda \right)+b_{\varphi }\left(\lambda \right),$$

where $a_{\omega }\left(\lambda \right),$ $a_{\gamma }\left(\lambda \right)\;$, and $a_{\varphi }\left(\lambda \right)$ represent the absorption coefficient of main medium particles (e.g., water, air), metal pipe wall, and other suspended particles, respectively, $b_{\omega }\left(\lambda \right)$ and $b_{\varphi }\left(\lambda \right)$ denote the scattering coefficient of main medium and other suspended particles, and λ indicates the wavelength of visible light [20,21,22].

Here, in Figure 1, the image transmission channel based on VLRC in the pipe is composed of a transmitter, a receiver, and several relay nodes. For each transmitter of the nodes, the light transmission will be influenced by factors such as the optical design of the convex lens and the design of the transmitter circuit [23]. Also, for each receiver of the nodes, the sensitivity of the light detector, the selection of filter and optical design of the convex lens can affect the transmission of the light. After considering the above factors, the image transmission channel based on VLRC can be described as the following equation:

(2) $$P_{n}\left(r\right)=P_{n-1}\left(t\right)\psi_{n-1}\left(t\right)\psi_{n}\left(r\right)exp\left[-c\left(\lambda \right)D\right]\times \frac{\pi R^{2}}{\pi {\left(Dtan\frac{\theta }{2}\right)}^{2}},$$

where $P_{n}\left(r\right)$ indicates the n  *th* received optical power, $P_{n-1}\left(t\right)$ represents the *(* n−1 *) th* transmitted optical power, $\psi_{n-1}\left(t\right)$ is the optical efficiency of *(* n−1 *) th* transmitter, $\psi_{n}\left(r\right)$ indicates the optical efficiency of n  *th* receiver, R denotes the convex lens radius of the receiver, and D and θ represent the transmission distance and divergence angle of the light, respectively.

In this research, to evaluate the VLRC-based image transmission performance within the pipe, an image quality evaluation framework is built based on the comparison between the final received images and original images. This framework focuses on evaluating image transmission performance through some typical image quality assessment algorithms such as mean square error (MSE), peak signal-to-noise ratio (PSNR), mean absolute error (MAE), and structural similarity index measure (SSIM), etc [24].

Here, we assume that the original two-dimensional gray image is $I=F\left(i,j\right)$, the size of image is M×N pixels, the received gray image is $I^{\prime }=F^{\prime }\left(i,j\right)$, and then the MSE and MAE can be described as follows:

(3) $$MSE=\frac{1}{M\times N}\times \overset{M}{\underset{i=1}{\sum }}\overset{N}{\underset{j=1}{\sum }}{\left[f\left(i,j\right)-f^{\prime }\left(i,j\right)\right]}^{2},$$

(4) $$\;MAE=\frac{1}{M\times N}\times \overset{M}{\underset{i=1}{\sum }}\overset{N}{\underset{j=1}{\sum }}\left|f\left(i,j\right)-f^{\prime }\left(i,j\right)\right|,\;$$

where 1≤i≤ *M,*  1≤j≤ *N*, $f\left(i,j\right)$ and $f^{\prime }\left(i,j\right)$ represent the pixel value of the original image and the final image at the pixel point of $\left(i,j\right)$, respectively, M and  N denote the width and height, respectively. MAE and MSE can reveal the pixel error between the original and final image. Besides, the PSNR can be defined as the following equation:

(5) $$PSNR=10lg\frac{{\left(L-1\right)}^{2}}{MSE},$$

where L is the maximum gray level of the image. For 8-bit images of 256 gray level, the value of L is considered as 256. The larger the PSNR value, the lower the signal loss rate during the signal transmission, the higher the image quality. Besides, as an image quality evaluation model for structural information measurement, the SSIM model can deal with the structure information of the image from three aspects: the brightness, contrast, and structure information of the image, and then synthesizes these three parameters to form the image quality evaluation index. The calculation formula is listed as follows:

(6) $$SSIM\left(I,I^{\prime }\right)=\;l\left(I,I^{\prime }\right)\times c\left(I,I^{\prime }\right)\times s\left(I,I^{\prime }\right),$$

(7) $$\;l\left(I,I^{\prime }\right)=\;\frac{2\mu_{I}\mu_{I^{\prime }}+K_{1}}{\mu_{I}^{2}+\mu_{I^{\prime }}^{2}+K_{1}},\;$$

(8) $$\;c\left(I,I^{\prime }\right)=\;\frac{2\sigma_{I}\sigma_{I^{\prime }}+K_{2}}{\sigma_{I}^{2}+\sigma_{I^{\prime }}^{2}+K_{2}},\;$$

(9) $$\;s\left(I,I^{\prime }\right)=\;\frac{\sigma_{II^{\prime }}+K_{3}}{\sigma_{I}\sigma_{I^{\prime }}+K_{3}},\;$$

where $l\left(I,I^{\prime }\right)\;$, $c\left(I,I^{\prime }\right)\;$, and $s\left(I,I^{\prime }\right)$ refer to the function of brightness, contrast, and structure information respectively, $\mu_{I}$ and $\mu_{I^{\prime }}$ denote the average brightness of the original image and the received image, $\sigma_{I}\;$, $\sigma_{I^{\prime }}\;$ and $\sigma_{II^{\prime }}$ indicate the brightness standard deviation and covariance of the original and received image, respectively, and $K_{1},\;K_{2}$ and $K_{3}$ are the constants. Usually, the larger the SSIM value, the higher the quality of the final image. This also means that the final image is more similar to the original image. In this article, the experiments on image transmission performance and image quality are based on these four evaluation models [25].

## 4. Analog Image Signal Relay Transmission (AISRT)

**Figure 2:**
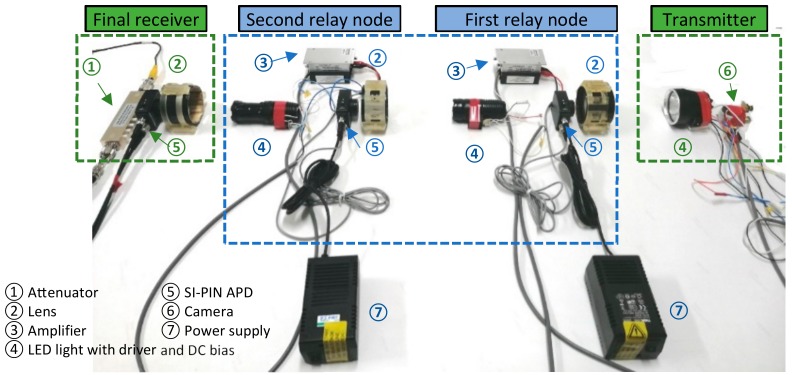
Entire architecture of AISRT system.

The analog video/image has been commonly applied in TV broadcast systems and surveillance equipment since it has some advantages such as more precise resolution and much easier realization. In this section, firstly, a fundamental composite video/image signal format is explained, then a basic optical system for AISRT has been established. The system for AISRT is demonstrated in Figure 2. The experimental verification on this system has been conducted which will be discussed in Section 6.

### 4.1. Composite Video/Image Signal

Currently, there are three major analog video signal standards in the world, including phase alternating line (PAL), national television system committee (NTSC), and séquentiel couleur à mémoire (SECAM) standards [26,27]. Among them, PAL is a color encoding system for analog television used in broadcast television systems in some regions such as Europe, Australia, and China. It can work at usually 625-line 50 fields (25 frames) per second. Another common color encoding system is NTSC. It has been mainly adopted by North America, South America, and Japan. It can work at 525-line 60 fields (29 frames) per second. The SECAM standard consists of three forms: SECAM-L, SECAM-B/G, and SECAM-D/K, which are mainly used in France, the Middle East, and Russia. Due to the relatively higher scanning rate (50 fields per second) of NTSC, in this research, a type of NTSC camera was used for the composite video analog signal transmission.

Composite video, also known as baseband video, is a traditional image data transmission method for the NTSC television signal. It can be transferred through a single analog waveform. The composite video contains color difference (hue and saturation of video), brightness, and synchronization signal. Especially, the synchronization signal can control the scanning of signals on the display including field sync signals, line sync signals, and line blanking signals.

In the waveform of the composite video signal, the brightness signal, and the synchronization signal are added together, which is called the luminance signal (Y). The hue and color saturation are converted into chromatic aberration signal through a certain conversion and then modulated on a color subcarrier. These modulated chromatic aberration signals can be considered as the chrominance signal (C), and its phase represents the color whereas its amplitude represents the color saturation.

### 4.2. Transmission System of Analog Image Signal Based on VLRC

As shown in Figure 2, in the system, a NTSC camera is used for in-pipe information acquisition. It possesses a 170-degree wide angle and 2.8 mm lens. The collected analog image signals are added with DC bias and then transmitted to the LED driver module. In this way, the image signal can be loaded onto the optical signal. Since the performance of LEDs has a great influence on the transmission quality of the system, a special XLamp XHP 70 LED array with 3930 lm output is selected as a suitable light source for the experiment [28]. When the image signal is transmitted to the channel in the form of the light beam, as mentioned in Equation (2), the beam has a divergence angle during the optical signal transmission. In order to increase the receiving power, at the first relay node, a convex lens with a diameter of 63 mm and a focal length of 150 mm is used to concentrate light so that the light can illuminate onto the photodetector at the focal length of the lens to avoid the divergence of light. Then we select Si PIN fixed gain detector PDA10A as the photodetector for each receiver in the system [29]. The wavelength response range of this detector is from 200 nm to 1100 nm, the active area is about 0.8 ${\mathrm{mm}}^{2}$. The signal bandwidth can reach almost 150 MHz and the maximum photoelectric conversion efficiency can even reach 0.44 A/W. After the conversion of the signal, the signal is amplified with a ZHL-6A-S+ amplifier and loaded onto the LED again [30]. Through the second signal relaying, the final receiver can receive the optical signal. After photoelectric conversion, the signal is adjusted by a KPATT2.5-90/1S-2N attenuator and eventually connected to the computer via a video capture card, so that the image captured by the camera is displayed on the computer screen in real time [31].

## 5. Digital Image Frame Relay Transmission (DIFRT)

In this section, the system architecture of DIFRT is presented. Typically, the function of each module is described in detail. Comparing with the previous AISRT approach, there are some additional steps in the image data processing before transmitting and after receiving using DIFRT method. These steps guarantee the transmission with high efficiency and low distortion.

### 5.1. System Architecture

**Figure 3:**
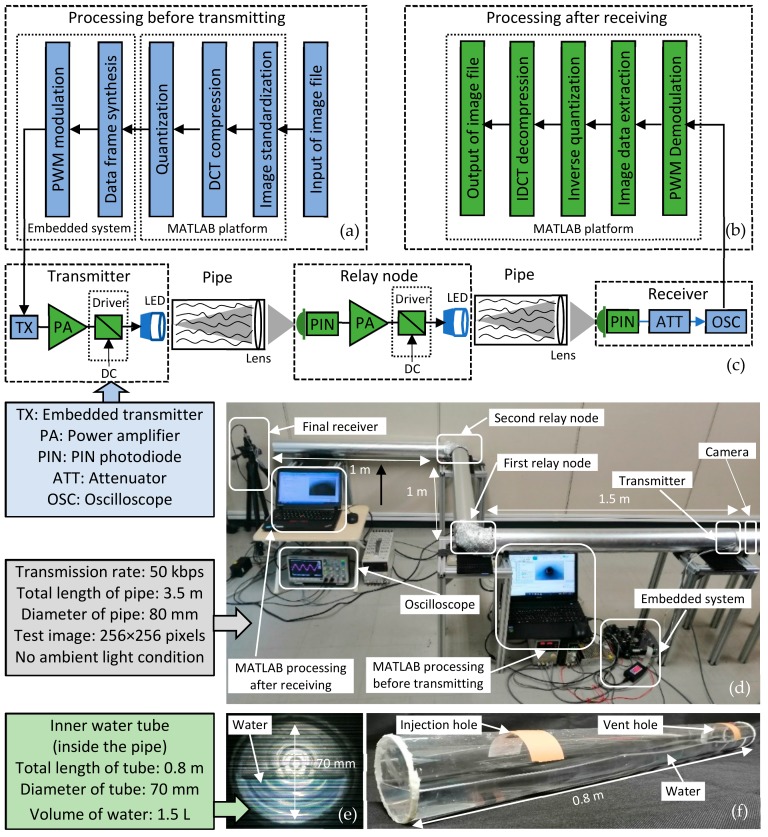
(**a–c**) Flow chart of digital image frame relay transmission (DIFRT), (**d**) setting up of DIFRT experiment, and (**e**,**f**) inner water tube.

The system can be conceptualized as the following major functional blocks as illustrated in Figure 3c. The whole system mainly consists of three components: the transmitter, the relay nodes, and the final receiver.

- *Transmitter*: The MATLAB platform can compress and quantize the original image captured by the camera, and also convert the image data into the special data frame. The data frame is transmitted to the embedded system (STM32F407ZGT) for processing. Through pulse width modulation (PWM) and data frame synthesis, this embedded system can modulate the data onto the LED (XHP-70) driver module in the form of binary bit data. Finally, the encoded binary bit information is transmitted in the form of visible light [32,33,34].
- *Relay node*: The relay nodes can receive and identify the data frame. After signal amplifying, the data frame is finally retransmitted to the next relay node.
- *Final receiver*: The photodetector (PDA10A2) firstly detects the square waves of visible light. After signal attenuation by the attenuator (KPATT2.5-90/1S-2N), the wave information is imported to the oscilloscope. Once the oscilloscope receives and saves a complete frame of image data, the data is transmitted to the MATLAB platform for offline demodulation, decompression, and reconstruction of the image.

Figure 3a,b also illustrate the flow chart of data processing before transmitting and after receiving.

Before the image transmission in the pipe, the image data should be processed by MATLAB and embedded system. Firstly, the image file captured by the camera is imported to MATLAB for image standardization. After this operation, the image can be converted to the standard grayscale image with a size of 256×256 pixels and a bit depth of 8 bits. Then this image is encoded and simplified by the DCT and quantization approach. Through data frame synthesis and modulation by the embedded system, the signal is sent out to the pipe channel by the transmitter. The processing after receiving can be roughly considered as the inverse method of the processing before transmitting. It includes demodulation, image data extraction, inverse quantization, and IDCT decompression. Figure 3d demonstrates the setting up of the experiment environment. This experiment will be described further in Section 6.3 and 6.4.

### 5.2. Image Compression and Decompression

The image compression and decompression are essential in digital image transmission. Because a large amount of uncompressed raw image data will take a lot of transmission time and even cause severe channel congestion. Therefore the image compression is necessary. In the research, the image is compressed and decompressed by the MATLAB platform. Moreover, the discrete cosine transform (DCT) and inverse discrete cosine transform (IDCT) methods are applied in image compression and decompression. DCT is suitable to compress data or images, and it can convert the spatial domain of signals into the frequency domain with good de-correlation performance. Generally, in the spatial domain, the correlation of image data is large. If the image data signal is converted from spatial domain to frequency domain, it will not affect the phase. Whereas the distribution of energy changes obviously from a relatively uniform distribution to concentration on a few low-frequency components. Some small components can be discarded directly without the degradation of image quality for efficient compression [35,36,37].

In MATLAB, image compression is processed with DCT function DCTMTX. The direct calculation of DCT is very complicated, hence we use the function BLKPROC to divide the original image into 4×4 pixel blocks in order to improve the conversion efficiency of DCT. Since the coefficient matrix of the DCT output owns a large frequency range, however, this frequency range does not substantially contain any image information. Therefore, the frequency coefficient can be simplified by quantization. Here, we adopt a 4×4 mask matrix to quantize the DCT frequency coefficient. At the receiver, the original image information can be finally reconstructed by inverse quantization and IDCT decompression.

### 5.3. Image Frame Realization

**Figure 4:**
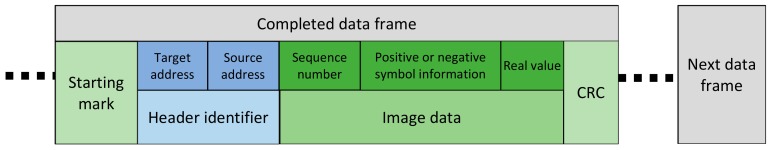
The structure of image data frame.

In the relay transmission mechanism, digital image signals are converted into a data frame in the transmission channel. The data frame format can be depicted as in Figure 4. It contains header identifier, image data, and cyclic redundancy check (CRC). In order to prevent information conflict and mutual-interference from each node, a source and target address are set as header identifier. The transmitter, first node, second node, and final receiver are set to 0xE0, 0xD0, 0xD1  and 0xF0,  respectively. Among the image data, we divide the information of the quantized frequency coefficient matrix into the sequence number, the positive or negative symbol information, and the real value. In this way, the image information is packed into data frames. The CRC can assist the receiver to identify the correctness of transmitted data and filter out useless information. At the final receiver, the image information will be extracted from the data frame and finally reconstructed.

In addition, due to the attenuation phenomenon resulted from absorption and scattering effects of the optical beam in the transmission channel, data error and loss of each frame are particularly common and inevitable. This effect can influence the quality of the reconstructed image. In this case, we will discuss in detail in Section 6.4.

## 6. Experimental Results and Discussion

To effectively analyze the image transmission characteristics of VLRC in the pipe, a series of experiments have been carried out in a pipe with two elbows (total length 3.5 m and 80 mm in diameter). The camera was mounted at a position 0.5 m from the end of the 1.5 m length straight branch pipe. The two VLRC nodes have been installed at the elbows of the pipe. Three convex lenses were placed at a distance of 150 mm from the receiver of each node. Moreover, we have designed two types of test environments. Usually, in order to safely enter the gas pipeline to detect damage, the pipe has to be opened in advance to release the gas to avoid accidents. In this research, we considered the gas pipeline as the empty pipe. For the water pipe, before being inspected by the mobile inspection system, valves were also cut off and even drained. However, some water was still inside the pipe. As depicted in Figure 3e,f, this research designed three 70 mm diameter 0.8 m length transparent sealed inner tubes which were filled with half of the water (nearly 1.5 L). Then we placed them inside the experimental pipe. We considered them as the water pipe environment. In these two scenarios, waveform tests on two signal transmission methods AISRT and DIFRT have been conducted. Four important coefficients MAE, MSE, PSNR, and SSIM which can describe the transmission efficiency, image transmission quality, and error were also recorded through the experiments. Furthermore, for DIFRT, the relationship between the BER and image quality has also been compared and analyzed. In particular, to guarantee the accuracy of experiments, the interference from ambient light has been strictly avoided [38,39,40].

### 6.1. Waveform Test on AISRT

**Figure 5:**
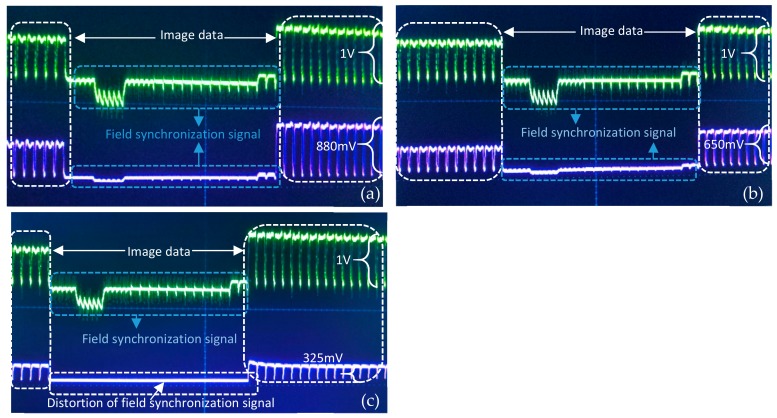
Waveform analysis of AISRT (**a**) waveform of first relay node, (**b**) waveform of second relay node, and (**c**) waveform of final receiver.

In this experiment, after signal amplification, the analog image signals were firstly loaded onto the LED driver and then transmitted through optical signals. After the relay transmission by two relay nodes, these optical signals were detected by the PIN photodiode of the final receiver. The digital signals were finally restored to images in the PC after photoelectric conversion by the photodiode and power adjustment by the attenuator. Figure 5 illustrated the waveform test on the AISRT in the water pipe. The purple wave in Figure 5a,b,c indicated the waveform of the video/image signal received by the first relay node, second relay node, and final receiver respectively. The green wave in each figure demonstrated the waveform of the original video/image signal sent by the transmitter. Generally, the analog video/image signal contained two components the field synchronization signal and image data. The signal could only be detected if the field synchronization signal was correctly judged. In Figure 5a,b, these field synchronization signals of first and second relay nodes could basically maintain synchronization with the original image signal from the transmitter, whereas sometimes the severe distortion of these signals could be observed just as depicted in Figure 5c. At this time, the distortion could induce the failure to recover from the video/image signal. Furthermore, the voltage peak-to-peak amplitude value of the original image signal was 1 V, however, this value decreased drastically during the transmission in the 3 m water channel. In the final receiver, the value reached an averagely 325 mV caused by the channel attenuation. This was also the reason why the image of the final receiver is dim. As conclusion, the short-range (<1 m) video/image transmission could be basically achieved by the AISRT method, nevertheless, for the longer distance, due to the severe distortion, this method could not be applied even using signal relaying.

### 6.2. Performance Evaluation of AISRT in the Different Mediums

As mentioned before, signal attenuation and blocking are very common when the signals are transmitted in the medium. As a kind of wireless waves, visible light also encounters signal attenuation in the medium, especially in liquids such as water. The scattering and absorption effects of photons in water are particularly obvious. This effect would even affect image transmission quality. In order to further investigate the transmission features in the different mediums, a series of important parameters such as MSE, MAE, PSNR, and SSIM were measured and analyzed. These four parameters were calculated with MATLAB based on grayscale images which converted from RGB images. The RGB images were actually transmitted through this AISRT system.

**Figure 6:**
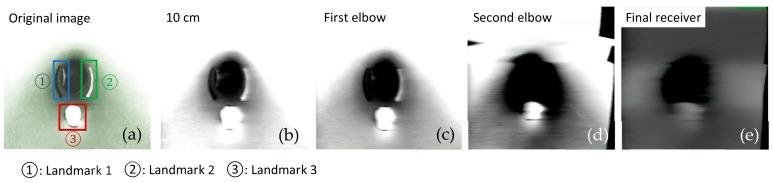
Analog image transmission with visible light in the empty pipe.

**Figure 7:**
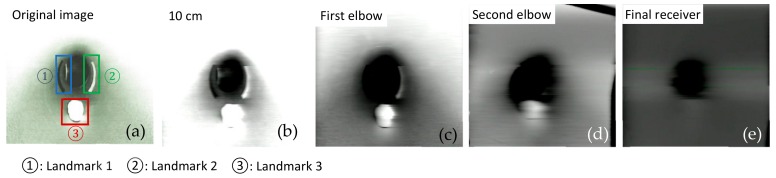
Analog image transmission with visible light in the water pipe.

In this test, we collected the RGB images at four receiving nodes: 10 cm distance from the transmitter, the first elbow, the second elbow, and the final receiving node outside the pipe. For each node, three image frames as experiment samples have been captured. As demonstrated in Figure 6 and Figure 7, three landmarks were labeled on the pipe wall [41,42,43]. In order to observe easily and truly reveal the deterioration of the image during the transmission, these three regular graphical landmarks 1, 2, and 3 were utilized to indicate three common situations, including corrosion, leakage, and blockage, respectively.

**Figure 8:**
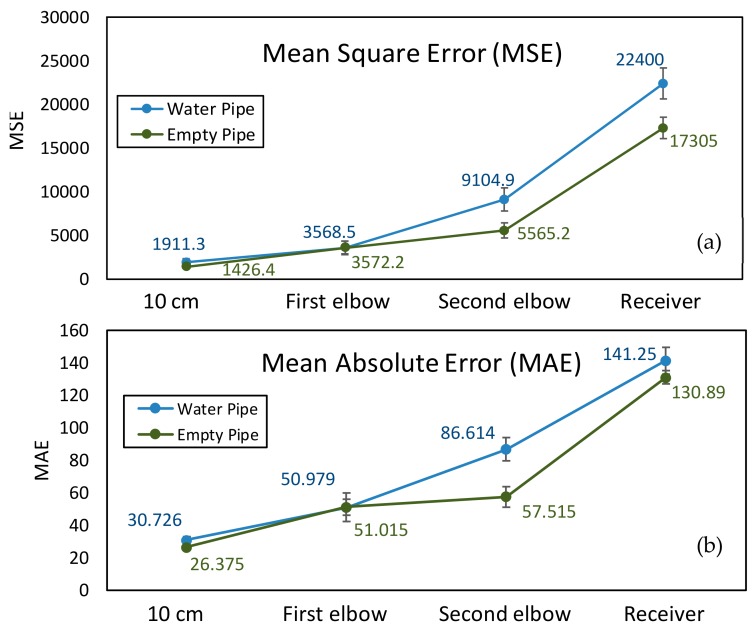
(**a**) MSE performance and (**b**) MAE performance for AISRT test.

**Figure 9:**
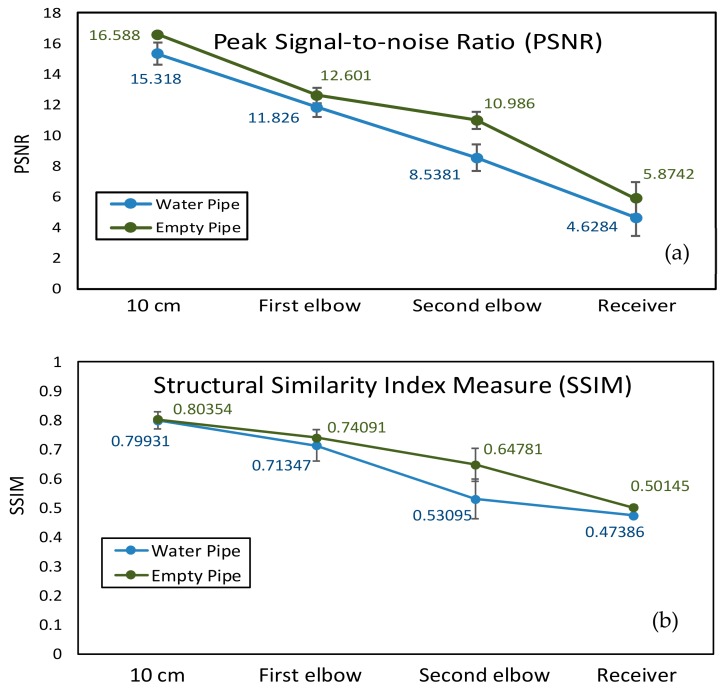
(**a**) PSNR performance and (**b**) SSIM performance for AISRT test.

As depicted in Table 1, landmarks 1 and 2 had the same real size L×W=6.5 cm×2 cm and same radian of 3π/4 from the camera vision in the pipe. These two landmarks were located on the pipe wall where is 10 cm from the end of the pipe and 15 cm from the camera. Landmark 3 had the size of radius=1.5 cm and height =1.2 cm. The location is almost 15 cm from the end of the pipe and 10 cm from the camera. From Figure 6 and Figure 7, we could observe that whether in the water or an empty pipe, the loss of image color is the most obvious. From 10 cm distance, the color has been lost. Also, the loss of image quality became extremely huger with the increase of communication distance. This loss could be mainly reflected in the three aspects: the noise, the structure, and the brightness of the image. The two typical parameters MSE and MAE could both reveal the error between the original image and the final received image. In particular, MAE could better reflect the actual situation of image abnormal error value, while MSE was more sensitive to abnormal values. In Figure 8a,b, with the increase of transmission distance in the water pipe, the noise of signal and pixel error became much larger. Therefore MSE increased significantly from 1911.3 to 22,400 and MAE rose from 30.726 to 141.25. The increase rate of MSE at the second elbow became higher in Figure 8a, which could be also observed from the image of Figure 7d to Figure 7e. The noise of the transmission channel would also have an impact on image sharpness and landmark recognition. PSNR was another important standard for measuring image quality. As mentioned before, the higher the value of PSNR, the higher the image quality. Within a distance of 10 cm between transmitter and receiver, PSNR could reach 16.528 and 15.318 in the empty and water pipe respectively. It appeared that the image signals were able to maintain good transmission condition whether in empty pipe or in water pipe within a short distance. However, as shown in Figure 9a, due to the scattering and absorption effects of water on the optical signal, PSNR of water pipe met a serious decline from 15.318 to 4.6284. The noise could also affect the recognition of target landmarks. In Figure 7b,c, the recognition of landmarks 1, 2 and 3 would be not difficult in the water pipe, in contrast, in Figure 7d the identification of the landmarks 1 and 2 is impossible when the PSNR reached almost 8.5381. The worst case is that we could not identify or judge any target landmark in Figure 7e because the structure of landmarks was missing in the image. In the empty pipe, the situation was better, however, the landmarks 1 and 2 could also not be found in Figure 6e. Although landmark 3 could be seen, the detail part of the image was completely missing with PSNR = 5.8742. Besides, we used another important parameter SSIM [44,45,46]. It could basically reveal the structure deterioration of image. Usually, the higher the SSIM value, the closer the image structure, brightness, and contrast of the received image is to the original image.

It can be observed from Figure 6 and Figure 7 that the structure and brightness of the images was apparently degraded in two transmission mediums. Especially, SSIM in the water pipe dropped obviously to 0.53095 at the second elbow in Figure 9b. In the situation of Figure 6b,c and Figure 7b,c, the image’s SSIM in the two transmission mediums could remain at nearly 0.71–0.80, whereas in the situation of Figure 6e and Figure 7e both SSIM decreased rapidly to approximately 0.50. In this case, structure deterioration occurred, and the image quality degraded significantly making it impossible to judge from the detailed structure and brightness of the image in the pipe [47].

### 6.3. Waveform Test on DIFRT

**Figure 10:**
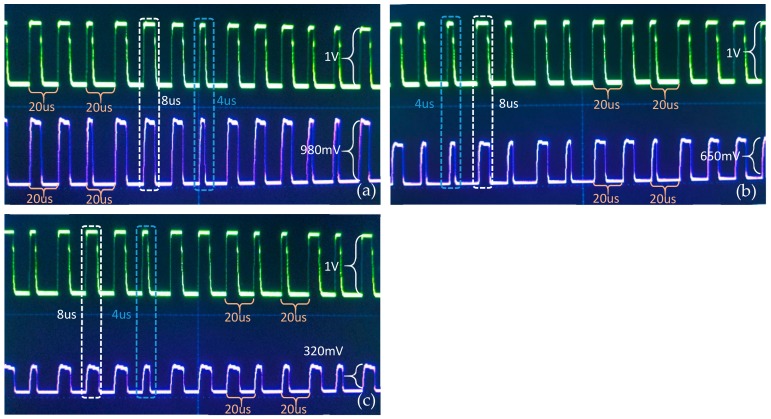
Waveform analysis of DIFRT (**a**) waveform of first relay node, (**b**) waveform of second relay node, and (**c**) waveform of final receiver (transmission rate=50 kbps).

The DIFRT method requires the camera to capture the in-pipe image. Then this image is converted to a grayscale image as the test original image. After compression, quantization, data frame synthesis, and modulation, these test images could be transmitted in the form of binary rectangular waves. As illustrated in Figure 10, part of the image data frame transmitted in the water pipe was recorded. The image transmission rate in this experiment was set 50 kbps. Moreover, the PWM method was used for modulation, the digital symbols ‘1’ and ‘0’ were defined based on the pulse width in one period. When a symbol ‘0’ was sent, it took a total of 20 μs, including 16 μs low level and 4 μs high level. For symbol ‘1’, it required 12 us low level and 8 us high level in the same period of 20 μs. The green waves indicated the original image data sent by the transmitter, while the purple waves in Figure 10a–c represent the signals received by the first, second, and final VLRC node, respectively. From observing Figure 10a, we could observe the purple wave was able to maintain a good synchronization and small distortion from the green wave. That means the light signal could keep relatively high transmission quality in the 1 m transmission channel although the effective voltage of the signal decreased slightly from 1 V to 980 mV. Compared with the original image signal, the purple waves in Figure 10b had some deformation and distortion due to the rising time delay of the second VLRC node and channel noise. The purple wave in Figure 10c demonstrated that a little waveform distortion from the receiver (second VLRC node) is the source of bit error rate which would have a certain influence on the transmission quality. Moreover, the effective voltage of the signal decreased to 320 mV.

).

### 6.4. Performance Evaluation of DIFRT with Different BER

In this test on DIFRT, the camera firstly captured three standard images inside the pipe. These images were converted into the test original gray image (size: 256×256 pixels, bit depth: 8 bits) in the PC. After DCT and quantization processing, the original digital image could be transformed into binary data in sequence. After data frame synthesis and PWM modulation, these binary data were loaded onto the light signal [48,49,50]. Through two relay transmission at a baud rate of 50 kbps, finally, the digital image frames were able to be reconstructed by decoding at the receiver. In this experiment, one important parameter BER was used to evaluate the performance of DIFRT. During the digital image transmission, the BER could inevitably exist, which had an immense impact on the final reconstruction of the digital image.

**Figure 11:**
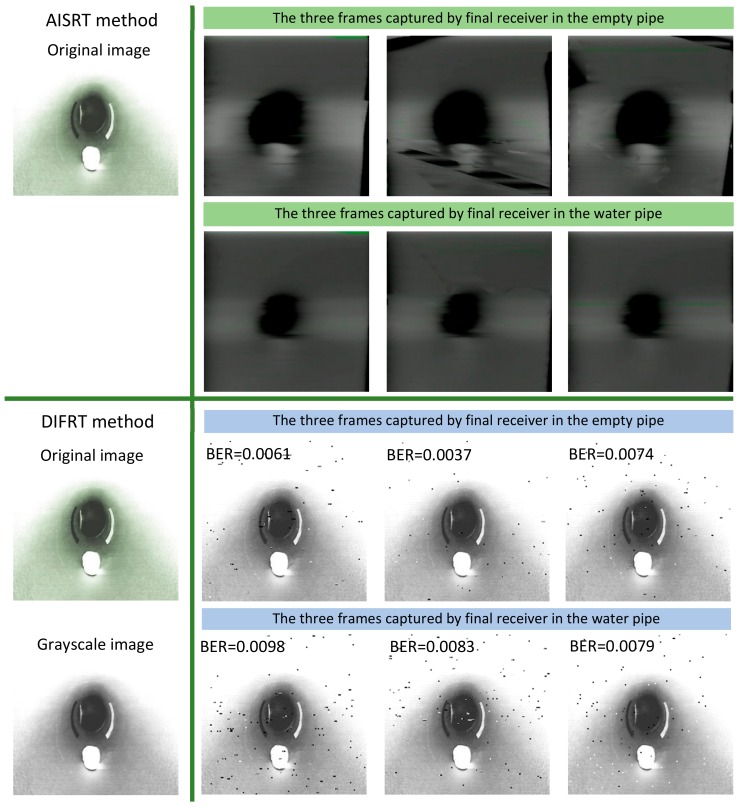
The performance comparison between DIFRT and AISRT.

As depicted in Figure 11, the leftmost column corresponds to the original images, and the right parts of this figure show the complete image received by the final receiver in the water and empty pipe after two signal relay transmission. For both AISRT and DIFRT methods, three image frames were captured and selected as experimental samples. It was evident that the image could be well reconstructed by the DIFRT method rather than the previous AISRT method. Figure 11 illustrates that DIFRT exhibited higher reconstruction performance considering the structure, brightness, similarity, and noise of final restored images. As further analyzed from Table 2, some statistical parameters such as PSNR, MSE, MAE, and SSIM of the reconstructed image in the final receiver have also been chosen as the criterion for evaluating image quality. Like AISRT method, the MATLAB calculation of these four parameters was also based on the 8 bit depth grayscale image, and therefore the performance comparison between DIFRT and AISRT was based on the same standard. In the water pipe, using DIFRT, the MSE could reach about 232.13–302.23, whereas the value was 21,992–22,829 with the AISRT method [51,52,53]. That means DIFRT has a stronger capability to deal with noise and interference caused by water absorption and scattering effects. This ability could be also reflected in the PSNR. The PSNR value after adopting the DIFRT method reached almost 24.473 which is almost five times the result obtained by utilizing the AISRT method. Although the SSIM value reached 0.90926 (in the water pipe) and 0.96146 (in the empty pipe), we could also observe that after the DIFRT approach was adopted, the reconstructed image structure and brightness was further improved compared with AISRT method. Besides, another conclusions could be obviously realized that the existence of a bit error rate would inevitably influence the whole image quality. In the empty pipe, with the BER ranging from 0.0037 to 0.0074, the PSNR and the SSIM value of the image were average 3.318 and 0.047 higher than the test value in the water pipe. That illustrated the water could in some degree reduce the transmission ability whether it was an analog signal or digital signal.

## 7. Conclusions and Future Work

In this article, we investigated the in-pipe image transmission based on the visible light relay communication (VLRC) technique. First, the optical transmission channel was introduced in order to explain the influences brought by the scattering and absorption effects in the pipe. Then, two types of image transmission approaches analog image signal relay transmission (AISRT) and digital image frame relay transmission (DIFRT) have been proposed and implemented. The AISRT system was built based on a complete optical transmitter, relay module, and receiver to achieve real-time image transmission. For the DIFRT system, apart from the basic transmission module, discrete cosine transform (DCT) compression and inverse discrete cosine transform (IDCT) decompression methods were adopted to realize more accurate and efficient transmission. Besides, a special data frame was designed to enhance the robustness and precision of in-pipe image transmission. To validate the feasibility and performance of VLRC-based image transmission systems, three typical experiments have been carried out in the empty and water pipe. Especially, for the water pipe, before inspection, the pipe was drained. However, some water remained in the pipe. Three transparent tubes which were filled with nearly 1.5 L water were inserted inside the experimental pipe to simulate the real water pipe environment. Firstly, the waveform test on the analog and digital image signal were conducted. The results revealed that, for the DIFRT method, the digital signals could basically achieve the relay image transmission in the 3 m length pipe channel, rather than the analog signals which had a huge signal distortion during the relay transmission. Secondly, the transmission performance in the different mediums using AISRT methods had been further evaluated. Analysis based on four parameters mean square error (MSE), peak signal-to-noise ratio (PSNR), mean absolute error (MAE), and structural similarity index measure (SSIM), we recognized that AISRT could not be selected as a suitable approach for VLRC-based image relay transmission. Finally, DIFRT-based image transmission with different bit error rate (BER) was tested. The conclusion could be achieved that digital image frame relay transmission was able to better overcome the effects brought by the absorption and scattering phenomenon and had relatively higher image transmission quality.

In summary, the DIFRT system based on VLRC technique demonstrates advantages in some aspects of the transmission range, the strong image reconstruction capability, and the transmission speed. Firstly, due to the signal relay, the image transmission could reach much longer range than the single point-to-point communication. Theoretically, if these technologies such as suitable re-modulation, signal amplifying, and filtering with relay node could be improved, the system could overcome the attenuation brought by the absorption and scattering effects. As a result, the transmission distance would become much longer. Besides, because of the relay node, the VLC system could be used in the non-line-of-sight (NLOS) transmission environment. Secondly, considering the image reconstruction ability, the image could be received by the final receiver and processed by MATLAB with less noise, more clear structure, and higher brightness. During the experiments, these three elements for evaluating in-pipe image quality could show a good image transmission performance when the DIFRT was adopted. Thirdly, in terms of transmission speed, due to the limitations of the transmitter hardware and pulse width modulation (PWM) method, the system transmission rate was set only 50 kbps in this research. This speed could basically satisfy the image transmission in the pipe. The transmission rate could be further improved by using other modulation methods or transmitters. Finally, in different mediums, the system was well verified. That means the system could be used in water or open air.

Although convincing results have been realized, this method still possesses some deficiencies that should be overcome in future work. These deficiencies are highlighted below:

- At present, the biggest and most difficult problem is the light alignment technology. Although visible light communication does not require high alignment requirements like laser communication, the alignment technology can severely affect the intensity of received optical power and finally influence the communication efficiency of the whole system. Currently, due to the difficulty in the light alignment, this system is unable to apply in the mobile robot system.
- During the transmission tests, the selection of the DIFRT method in this research has not been appropriately justified. Since the BER still exists during the transmission, some other modulation/demodulation should be also sufficiently considered. In addition, currently, the offline process of image compression and decompression make the system unable to be used in the small pipe.
- Currently, this DIFRT system cannot transmit and receive RGB images. There are mainly two important reasons for this: (1) The calculation of MSE, MAE, SSIM, and especially PSNR is based on 8 bit depth grayscale image. In order to compare transmission performance between the DIFRT and AISRT methods based on these four parameters, a standard grayscale image is required. (2) RGB images are considered as 24 bit depth images, which means that each pixel from the image has a maximum $2^{24}$ value, whereas each pixel of a grayscale image has a $2^{8}$ value. This big data scale of RGB pixels will increase the transmission task even if these data are compressed and transformed into a binary data. In the future, the effective transmission of RGB images using VLRC technique requires more in-depth research.
- To realize efficient image transmission within a pipe over a much longer distance is still a difficult task. Currently, the maximum point-to-point transmission distance achievable by the DIFRT method is almost 1.7 m, therefore, the maximum distance can reach a total of 5.1 m with two relay nodes in the pipe. In theory, the transmission range can be expanded by increasing the number of relay nodes (follower robots). However, as the BER increases, the DIFRT system will be restricted in a certain distance. The limiting number of relay nodes should be further further investigated. In addition, increasing the number of relay nodes will enhance the risk of forming a robot chain system. Since optical signal distortion and attenuation such as signal amplitude and noise occur during the transmission, the utilization of the suitable wave-shaping module and signal amplifier for each relay node can assist the whole system to achieve longer transmission with fewer follower robots.

**Figure 12:**
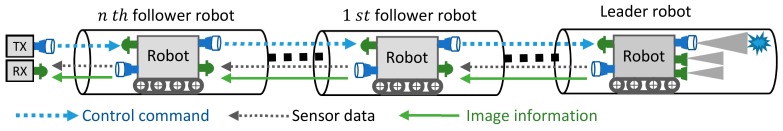
The whole architecture of the RCS.

If the above deficiencies can be solved, a high-quality, long-range, high-efficiency image transmission technique can be implemented on pipe inspection robots. The RCS is capable of operating at much longer distances and sending back much higher quality image information from the pipes. This image information will help operators analyze the internal damage of the pipeline. It will be also conducive to pipes’ timely maintenance, repair, and other regular operations. Combined with the previous technique, the possible architecture of RCS is illustrated in Figure 12, in which multiple relay nodes (from to ‘1 st follower robot’ to ‘*n* t*h* follower robot’) are connected in a ‘chain’ and establish a bidirectional relay system. In the bidirectional transmission mechanism, the sensor data (e.g., air pressure, concentration, humidity, and temperature) and image data are transmitted to the external display terminal through the ‘follower robots’. The control commands are sent back to the ‘leader robot’ from the external control terminal. In the future, different types of robot chain systems will be developed and finally applied to some other complex environments such as tunnels, mines and underwater, etc.

## Figures and Tables

**Table 1:** The characteristics of landmarks in the pipe.

Land Mark	Pipe Information	Pipe Image	Actual Size	L × W (cm)	θ (rad)	Color
①	corrosion	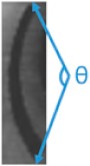	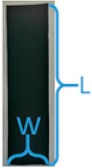	6.5 × 2	$\frac{3}{4}$π	black
②	leakage	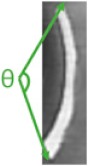	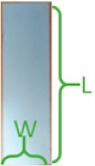	6.5 × 2	$\frac{3}{4}$π	white
③	blockage	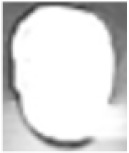	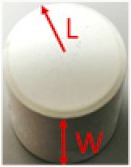	1.5 × 1.2		white

**Table 2:** Comprehensive performance comparison between DIFRT and AISRT for the final receiver.

Type	Scenarios	BER	MSE	PSNR	MAE	SSIM
AISRT	Empty pipe		17305	5.8742	130.89	0.50145
			17548	4.5832	141.71	0.47197
			16173	4.6725	140.48	0.48723
	Water pipe		22400	4.6284	141.25	0.47386
			22829	4.5459	143.05	0.41676
			21992	4.7082	139.92	0.45027
DIFRT	Empty pipe	0.0061	158.47	26.131	0.77844	0.94118
		0.0037	76.156	29.314	0.42062	0.96146
		0.0074	162.54	26.021	0.82405	0.92654
	Water pipe	0.0098	302.23	23.327	1.5842	0.88439
		0.0083	276.87	23.709	1.3531	0.89202
		0.0079	232.13	24.473	1.0764	0.90926

## Abbreviations

The following abbreviations are used in this manuscript:

RF	Radio Frequency
IR	Infrared Radiation
LOS	Line of Sight
NLOS	Non Line of Sight
CCTV	Closed Circuit Television
WRC	Wireless Relay Communication
VLC	Visible Light Communication
VLRC	Visible Light Relay Communication
DCO-OFDM	Direct Current Biased Optical- Orthogonal Frequency Division Multiplexing
PWM	Pulse Width Modulation
RCS	Robot Chain System
BER	Bit Error Rate
CRC	Cyclic Redundancy Check
AISRT	Analog Image Signal Relay Transmission
DIFRT	Digital Image Frame Relay Transmission
DCT	Discrete Cosine Transform
IDCT	Inverse Discrete Cosine Transform
LED	Light Emitting Diode
NTSC	National Television Committee
PAL	Phase Alternating Line
SECAM	Séquentiel Couleur à Mémoire
MAE	Mean Absolute Error
MSE	Mean Square Error
PSNR	Peak Signal-to-Noise Ratio
SSIM	Structural Similarity Index Measure
